# Media devices in pre-school children: the recommendations of the Italian pediatric society

**DOI:** 10.1186/s13052-018-0508-7

**Published:** 2018-06-14

**Authors:** Elena Bozzola, Giulia Spina, Margherita Ruggiero, Luigi Memo, Rino Agostiniani, Mauro Bozzola, Giovanni Corsello, Alberto Villani

**Affiliations:** 1 Pediatric and Infectious Diseases Unit, IRCCS Bambino Gesù Children Hospital, Rome, Italy; 2 Pediatric Department, S. Martino Hospital, Belluno, Italy; 3 Department of Pediatrics, Ospedale del Ceppo, Pistoia, Italy; 4 Internal Medicine and Therapeutics Department, Pediatrics and Adolescentology Unit, University of Pavia, Fondazione IRCCS San Matteo, Pavia, Italy; 5 Operative Unit of Pediatrics and Neonatal Intensive Therapy, Mother and Child Department, University of Palermo, Palermo, Italy

## Abstract

**Background:**

Young children are too often exposed to mobile devices (MD) and most of them had their own device. The adverse effects of a early and prolonged exposure to digital technology on pre-school children has been described by several studies.

Aim of the study is to analyze the consequences of MD exposure in pre-school children.

**Methods:**

We analyzed the documented effects of media exposure on children’s mental and physical health.

**Results:**

According to recent studies, MD may interfere with learning, children development, well being, sleep, sight, listening, caregiver-child relationship.

**Discussion:**

Pediatricians should be aware of both the beneficial and side effects of MD and give advice to the families, according to children’s age.

**Conclusion:**

In according to literature, the Italian Pediatric Society suggest that the media device exposure in childhood should be modulated by supervisors.

## Background

Technology progress is leading to an increase of media use including broadcast and interactive media among people, from toddlers to adults. In particular, broadcast media include television and movies while interactive ones include social media and video games.

Nowadays, even pre-school children are growing up in environments satured with internet, computer and video games that strongly catch their attention.

According to an American survey, the percentage of children aged 0–8 years using a mobile device increased from 38% in 2011 to 72% in 2013. Focusing on 2 years old children the increment was even higher, moving from 10% in 2011 to 38% in 2013 [1].

Smartphones and tablets are the most frequently used device (51 and 44% respectively).

The mobile app used (educational games and creative apps) has been also documented. Fully half (50%) of all children ages 0 to 8 have used mobile apps, up from just 16% in 2011. In particular, these devices are used even by younger kids. In fact, 13% of children younger than 1 year used educational games and 19% creative apps [1].

A cross-sectional study on technology habits of 350 children aged 6 months- 4 years produced similar results. In details, almost all children (96.6%) used mobile devices (MD). Most of kids (92,2%) started using a mobile media device before the age of 1 year. And at the age of 2 years, most children used a device daily [2].

The adverse effects of a early and prolonged exposure to digital technology on pre-school children has been described by several studies [3–5].

Among the side effects, the most reported are interferences on neurocognitive development, learning, well-being, sight and listening, metabolic and cardiological functions.

Recent studies have demonstrated that parental mobile devices usage influences child safety, emotional well-being and family interactions. In fact devices distract parents-children dyad from face-to-face interactions, having a great impact in cognitive, language, and emotional development [6].

Moreover, MD may be used to placate or distract children or as means to manage children’s behavior. Studies revealed that parents often give children devices when doing house chores, to keep them calm in public places, during meals and/or at bedtime to put their child to sleep [2, 7, 8].

Pre-school children have often at least one screen-based electronic device in their own bedroom [9].

In Italy, few data are available on media use in children. A recent survey described that 20% of children used a smartphone for the first time during his first year of life. Moreover, 80% of children from 3 to 5 years old is able to use their parent’s smartphone. In addition, parents often use media as pacifiers, giving mobile devices to their child to keep them calm during the first (30%) and the second (70%) year of life [10].

### Aim

Aim of the study is to analyze the evidences of MD in pre-school children.

## Methods

For the purpose of the study, we investigated both beneficial and negative effects of media on children’s mental and physical health in order to discuss age-appropriate child’s exposure to media.

## Results

### Learning

According to recent studies, touch screen usage may interfere with infant and toddler learning development. In fact, young children need direct first-hand experience with materials and equipment that challenge their thinking and problem solving skills. Moreover, no substitute for direct interaction with parents has been found [11].

On the other hand, children younger than 3 years old can learn words through video if specific conditions are fulfilled. In details, children would be able to learn from video when the experimenter/parent/caregiver provide additional verbal and non-verbal information during the live action sequences [12]. In particular, speaker’s eye gaze is an important communicative signal of non-verbal caregiver-children interaction [13].

Mobile phones could be a tool to reinforce what children are already learning at school. In particular, using well-designed educational apps promote learning among preschool and early-elementary-aged children [14]. Unfortunately, most of the downloaded apps are not designed for a dual audience (both parent and child), targets only rote academy skills and are not based on established criteria from developmental specialist or educators.

Better policies for the evaluation of content in various app stores could be a means to create high quality educational apps for kids [14].

### Development

Children’s development is also influenced by background television: it has been proven to have negative effects in young children’s brain development, reducing the amount and quality of parents-children dyad interactions [5].

As reported by Pagani et al. high rates of screen time are related to decrements in math and attention scores but also to peer rejection experiences [4].

Drawing is an activity that allows child thinking and creation of his own prospective of things [15, 16]. In this context, drawing apps can play a positive role in child development [17]. Drawing apps can be used as a supplement to traditional crayons and chalk as they are safe and easily to use [18].

Media devices use has been associated to task inefficiency, loss of attention, and safety hazards [3]. Thus, media use by children and toddlers could have positive effects only with right contents and parents’ interaction presence [19].

### Well-being

Electronic media use during early childhood for more than 2 h per day has been linked to increased weight status and to behavioral problems. In particular, well-being can be conceptualized as constituting positive and adverse psychological and social attributes and behaviors. Poor levels of well-being during early childhood are associated with later outcomes, such as depression and hostile and aggressive behavior [20]. Television viewing and videogames are related to increased rates of obesity, sedentary behaviors during childhood and wrong dietary behaviors [21].

Some evidence suggested that there is an association between physical discomfort and tablets usage, especially involving neck and shoulders. In particular, children as young as 8 years old are being treated for headaches, neck and shoulder pain and poor posture as they spend more time with screens, including mobile phones. The Australian Physiotherapists Association agreed that there is an emerging of physical problems among children related to an abuse of media usage: the right balance between screen time and physical activity should be obtained [22].

### Sleep

Media usage may interfere with sleep quality through the increase of psychophysiological arousal caused by stimulating content watched, or through bright light exposure [23]. Bright light may impact sleep by delaying the circadian rhythm when exposure takes place in the evening and also by causing an immediate activation in itself [24, 25]. According to the model exposed, sleep may also be negatively impacted by electromagnetic radiation [23]. Another mechanism relates to physical discomfort, such as muscular pain and headache, caused by prolonged media use and inappropriate neck posture during activities such as game playing [26]. Furthermore, screen use over two hours per day is significantly associated with long sleep onset latency especially in children that use more devices at the same time compared with those using only one device [27].

A recent study, conclude that among children aged between 1 to 4 years old, the presence of a television in the bedroom is associated with significantly reduced sleep quality, sleep terrors, nightmares, and sleep talking [28].

### Sight

A reduced blink rate during continuous smartphone use causes faster evaporation of the tear film, which may then lead to dry eye disease. Moreover, smartphones are used with short watching distances due to their small LED screens, thus inducing ocular fatigue, glare, and irritation [29].

Excessive smartphone use at a close reading distance might influence the development of a condition called acute acquired comitant exotropia that is an unusual presentation of exotropia in older children. It can potentially be induced by an increased tonus of medial rectus muscles resulting from disrupted accommodation and vergence by video Display work. In these cases, avoid smartphone usage can decrease the amount of esodeviation leading to successful management of residual exotropia and restoration of binocularity [30].

### Listening

The early and prolonged exposition of eardrums to intense levels leads to a dangerous sound immersion without a break period for ears. In fact, in this context, speech and language development may be compromised. Difficulties in socializing, communicating and interacting with other kids may be possible side effects [31].

### Caregiver-child interaction

Early and adequate children-caregivers interactions contribute to behavioral and neurocognitive system development. Eye contact, mutual gaze, or joint visual attention between children and caregivers during emotionally charged discussion are related to autonomous regulation and development of healthy attachment relationships [32]. Media use causes fewer verbal and nonverbal interactions between parents and children dyad, parent-child conflict oppositional defiant disorder and callous unemotional behaviors [32–34].

In this scenario, parents’ background television and mobile devices usage, distracts from parent–child interactions, child play and may affect child cognitive development and concentration [5, 35].

## Discussion

Pediatricians have an important role in advising the exposure to MD in childhood. Nevertheless, according to a recent study, only 16% of pediatricians ask families about their media use and only about 29% of parents report relying on their pediatrician for advice about media [36].

On the contrary, pediatricians should explain to families both the beneficial and side effects of mobile device according to children’s age. Devices can be used for entertainment, social support, or access to educational materials for children but they may cause side effects when not well used.

Pediatricians should discuss with parents about healthy consequences related to MD usage such as inadequate sleep, reduced physical activity, parents-child interaction, brain development. In particular, pediatricians have a key role in educating parents about the importance of hands-on, unstructured, and social play to build language, cognitive, and social-emotional skills, identifying areas in which good health and wellness can be enhanced. Therefore, help parents in facing challenges, such as setting limits and finding alternate activities to calm their children.

Pediatrician and families should create a network to manage the digital landscape involving their children.

## Conclusion

In conclusion, in according to the American Academy of Pediatrics and with the Australian guidelines we suggest that the media device exposure in childhood should be modulated on the basis of the clinical evidence [3, 9].

In details:

- we recommend no media devices use:

- in children under 2 years of age
- during meals
- at least for 1 h before bedtime
- in case of fast-paced programs, apps with distracting or violent content
- as a limit pacifier, to keep children quiet in public places.

b) we suggest to limit media exposure:

- to less than 1 h per day in children aged 2–5 years,
- to less than 2 h per day in children aged 5–8 years
- to high-quality programming
- just in presence of an adult. Children should share the use of media devices with caregiver in order to promote child’s learning and interactions. In a world where children are “growing up digital”, parents play an important role in teaching them how to use technology safely. Families should monitor children’s media content and apps used or downloaded.
- to apps tested by a care-giver before the child usage. More than 80,000 apps are labeled as educational, but few researches have demonstrated their actual quality. Parents should check age-appropriate apps, games and programs to make the best choices for their children. To make sure of the quality of media used, parents can consult with pediatricians on what kids are viewing and about any issues associated with media

Finally, pediatricians must remember to families that parents should be a good role model: children are great mimics. For this reason, parents have to limit their own media use. A more connection with children will be obtained interacting, hugging and playing with them rather than using media. Families don’t have to use media as an emotional pacifier because it will limit children development of his own emotion regulation.

Parents and children proactive interaction is likely the best approach.

## Abbreviation

MD: Mobile device
